# Single Nucleotide Polymorphisms in Pathogen Recognition Receptor Genes Are Associated with Susceptibility to Meningococcal Meningitis in a Pediatric Cohort

**DOI:** 10.1371/journal.pone.0064252

**Published:** 2013-05-14

**Authors:** Gijs Th J. van Well, Marieke S. Sanders, Sander Ouburg, Vinod Kumar, A. Marceline van Furth, Servaas A. Morré

**Affiliations:** 1 Department of Pediatric Infectious Diseases, Immunology and Rheumatology, VU University Medical Center, Amsterdam, The Netherlands; 2 Department of Pediatrics, Maastricht University Medical Center, Maastricht, The Netherlands; 3 Department of Medical Microbiology and Infection Control, Laboratory for Immunogenetics, VU University Medical Center, Amsterdam, The Netherlands; 4 Department of Human Genetics, University Medical Center Groningen, Groningen, The Netherlands; 5 Institute of Public Health Genomics, Department of Genetics and Cell Biology, Research Institute: School for Oncology & Developmental Biology, Faculty of Health, Medicine & Life Sciences, Maastricht University Medical Center, Maastricht, The Netherlands; Oxford University, Viet Nam

## Abstract

Bacterial meningitis (BM) is a serious infection of the central nervous system, frequently occurring in childhood and often resulting in hearing loss, learning disabilities, and encephalopathy. Previous studies showed that genetic variation in innate immune response genes affects susceptibility, severity, and outcome of BM. The aim of this study is to describe whether single nucleotide polymorphisms (SNPs) in pathogen recognition gene products are associated with susceptibility to develop BM in single genes analysis as well as SNP combinations. Genotype frequencies of seven SNPs, in five immune response genes encoding for Toll-like receptors (TLRs), nucleotide oligomerization domain (NOD) proteins and caspase-1 (CASP1), in 391 children with meningococcal meningitis (MM) and 82 children with pneumococcal meningitis were compared with a large cohort of 1141 ethnically matched healthy controls. Carriage of *TLR4* +896 GG mutant predisposed to susceptibility to develop MM (*p* = 1.2*10^−5^, OR  = 9.4, 95% CI  = 3.0–29.2). The *NOD2* SNP8 mutant was significantly more frequent in MM patients compared to controls (*p* = 0.0004, OR  = 12.2, 95% CI  = 2.6–57.8). Combined carriage of *TLR2* +2477 and *TLR4* +896 mutants was strongly associated with MM (*p* = 4.2*10^−5^, OR  = 8.6, 95% CI  = 2.7–27.3). A carrier trait of *TLR4* +896 and *NOD2* SNP8 mutants was also strongly associated with susceptibility to develop MM (*p* = 4.2*10^−5^, OR  = 10.6, 95% CI  = 2.9–38.6). This study associates SNPs in *TLR4* and *NOD2* with susceptibility to develop MM.

## Introduction

Susceptibility to infections is determined by genetic variation in human populations as can be concluded from genetic epidemiology studies. An important challenge is identifying the responsible genes and translating these findings into biological mechanistic explanations [1], [2]. Bacterial meningitis (BM) is a severe infectious disease of the central nervous system (CNS). It occurs relatively frequent in childhood and often affects hearing and learning abilities [3], [4]. Immune responses to BM causing pathogens are primarily aimed at eliminating bacteria from the CNS by recognition of microbial ligands and subsequent triggering of production of specific cytokines, but these cytokine responses also contribute to collateral damage to healthy neuronal tissue and thus adverse outcome [5]. Genetic variation in genes encoding for pathogen recognizing receptors (PRRs), such as Toll-like receptors (TLRs) and nucleotide oligomerization domain (NOD) like receptors (NLRs), can lead to enhanced or decreased inflammatory responses in several cell types such as macrophages and epithelial cells [6]. Microglia, the resident macrophages inside the CNS, and neuro-epithelial cells also express TLRs and NLRs. Thus, genetic variation in these receptors might influence susceptibility, severity and outcome of BM.

Several genetic association studies have shown that single nucleotide polymorphisms (SNPs) in innate immunity genes were associated with susceptibility to meningococcal and pneumococcal disease, including cases of meningitis [7]–[9]. We previously described that carriage of the *TLR9* +2848 A allele protects against BM [10]. Severity analysis revealed that SNPs in *TLR2*, *TLR4* and *TLR9* are associated with hearing loss in survivors of BM [11]. In this study, we compared genotype distributions in a larger group of BM patients with a big cohort of healthy controls in order to discover susceptibility genes. We focused on innate immune response genes in BM caused by *Neisseria meningitidis* and *Streptococcus pneumonia*e, the two most common causing pathogens of BM in the world [3]. We selected seven SNPs in five immune response genes, based on BM pathogenesis, which usually starts with nasopharyngeal colonization and subsequent epithelial disruption by bacterial components, enabling these bacteria to enter the bloodstream where they replicate and cause bacteraemia, and next, might cross the blood-brain barrier (BBB) and multiply in the subarachnoid space [12]. The immune response inside the CNS upon bacterial entrance starts with pathogen recognition by microglia and astrocytes and by non-neuronal structures in direct contact with the cerebrospinal fluid (CSF), such as dendritic cells and macrophages, all expressing PRRs. PRR activation triggers an intracellular signaling cascade resulting in the transcription of pro-inflammatory cytokines and chemokines, also inside the CNS [6]. Cytokine induced increased permeability of the BBB and chemokine induced influx of inflammatory cells from the bloodstream into the CNS result in enhancement of the local inflammatory response inside the brain. The clinical consequence is brain edema, raised intracranial pressure, infarction and neuronal injury [12]. The ability of a host to sense microbial CNS invasion and to respond appropriately to control the local infection is essential for killing these microbes but the inflammatory response also results in the production of several cytotoxic mediators responsible for damage to healthy neuronal cells and thus for adverse disease outcome [12].

The potential relevance of the studied SNPs in BM have been described in detail before [11]. In short, TLR2 and TLR4 are PRRs located on the surface of immune cells and recognize cell wall components of Gram-positive and Gram-negative bacteria respectively. Animal data have shown that deficiency of TLR2 and TLR4 leads to reduced bacterial clearance from the CNS in response to *S. pneumoniae* infection [13]. NOD1 and NOD2 recognize degradation products of peptidoglycan (PGN). Murine astrocytes and microglia express robust levels of NOD2 after exposure to both *N. meningitidis* and *S. pneumonia*e [14], [15]. Cysteine-dependent aspartate-directed protease (Caspase) plays an essential role in apoptosis and cytokine production. CASP1 levels are upregulated in the CSF of patients with BM and correlate with clinical outcome, and *CASP1* −/− mice, intracerebrally infected with *S. pneumonia*e, show a significantly attenuated increase of IL-1β, lower CSF leukocytes, and an improved clinical status [16].

The aim of this study was to identify associations of genetic variation in the aforementioned seven single and combined SNPs with susceptibility to BM.

## Materials and Methods

### Patients

**Figure 1:**
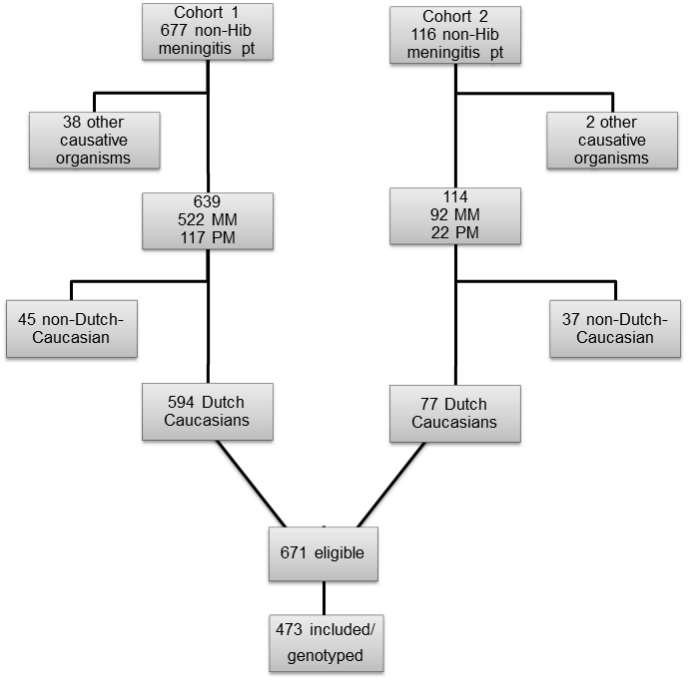
Flow chart of patient inclusion in this study.

*Figure 1* shows a flow chart of patient inclusion in this study. All patients were selected from data on bacterial CSF isolates of the Netherlands Reference Laboratory for Bacterial Meningitis. Only Dutch Caucasian survivors of meningococcal meningitis (MM) and pneumococcal meningitis (PM) were asked to participate in this study. The original cohort consists of children born between January 1986 and December 1994 who survived BM between January 1990 and December 1995 [17]. The validation cohort consists of children born between January 1993 and December 1999 who suffered from BM between January 1997 and December 2001 (work in progress). Clinical characteristics of both cohorts were comparable and no significant differences in genotype distributions were observed between both cohorts.

Six hundred seventy one (671) eligible patients were asked to return a sterile swab after collecting their buccal DNA. Of these patients and their parents/caretakers/guardians, a total of 473 (70%) have given written informed consent to publish these case details and returned a buccal swab. The cohort consisted of 391 former MM patients and 82 former PM patients. The median age of all patients at the time of infection was 2.2 years of age (range 0.1–9.5) and 56% were male. Children with ‘complex onset’ of meningitis (defined as meningitis secondary to immune deficiency states, cranial trauma, CNS surgery, and CSF shunt infections, meningitis in the neonatal period) or relapsing meningitis were not included. The Medical Ethical Committee of the VU University Medical Center approved this study.

### Controls

Controls were unselected, unrelated blood bank donors and population controls, drawn from volunteers at the University Medical Centers of Utrecht and Groningen. It consists of 493 (43%) females and 648 (57%) males ranging from 18 to 70 years old. All controls were from The Netherlands descent and at least three of their four grandparents were also born in The Netherlands. Since these individuals are mostly volunteers from blood bank without any registered complications, we considered them as “healthy”. Written informed consent was obtained from all subjects, with approval of the VU University Medical Ethical Committee, the University of Utrecht Medical Ethical Committee, and the University Medical Center Groningen Medical Ethical Committee. Genotype data for 1141 control samples were extracted from Immunochip platforms using PLINKv1.07 [18], [19].

### DNA Isolation

DNA was isolated from the buccal swabs using the following procedure: after addition of 250 µl 10 mM Tris-HCl (pH 7.4) the sample was heated at 96°C for 10 minutes. After mixing for 10 seconds the swabs were removed and the sample was centrifuged for a few seconds (14000 rpm). In controls, venous blood (5–10 ml) was drawn and genomic DNA was isolated using standard protocols and 5–100 ng of genomic DNA was used each cycle of genotyping.

### Genotyping

All case samples were genotyped for the *TLR2* +2477 G>A (rs5743708), *TLR4* +896 A>G (rs4986790), CARD4/*NOD1* +32656 C>A (rs6958571), *CARD15/NOD2* +2209 A>T (rs2066844), *CARD15/NOD2* +2722 G>T (rs2066845) *CARD15/NOD2* +3020 ins C (rs2066847) and *CASP1*-8404 A>G (rs2282659) SNPs by real-time polymerase chain reactions (RT-PCR) using the TaqMan AbiPrism® 7000 Sequence Detection System (Applied Biosystems, UK) and the LightCycler® 480 System (Roche Applied Science, US), using standard manufacturer's protocols. Results were checked twice by two independent researchers. Control samples were genotyped using the Immunochip according to Illumina's protocols and NCBI build 36 (hg18) mapping was used to assign SNP location (Illumina manifest file Immuno_BeadChip_11419691_B.bpm).

### Statistics

Genotypes were compared between cases and controls for MM and PM separately and for all cases of BM together. Hardy-Weinberg tests were used to check the observed genotype distributions in the control population. Fisher's exact and Chi-^2^ tests in case of sample sizes >5 were used to calculate statistical significance of differences in genotype frequencies between cases and controls. For statistical analysis, SPSS Statistics 17.0 (IBM Corporation, Somers, NY and GraphPad Prism 5 were used. *P*-values <0.05 were considered statistically significant. Subsequently, the single genotypes were used to define carrier traits. With carrier trait analyses we investigated combinations of SNPs. We studied the implication of the combined effect of individual SNPs on susceptibility to BM. Based on associated biological pathways and guided by the results of the single gene associations we tested which combinations of two SNPs showed an enhanced statistical association. After correction for multiple testing according to Holm-Bonferroni, *p*-values <0.0006 were considered to be statistically significant in the trait analyses.

## Results

### Hardy-Weinberg tests

Genotype distributions of all SNPs in the control groups were in Hardy-Weinberg Equilibrium (HWE). In the cases *TLR2*, *TLR9*, *NOD1*, and *CASP1* SNPs were in Hardy-Weinberg Equilibrium (HWE), *TLR4* and *NOD2* SNPs were not in HWE.

### Single gene analysis

Genotype frequencies of BM patients were compared to those in controls and MM and PM patients were also separately compared to controls in order to discover associations between SNPs and susceptibility to a specific pathogen. The results are summarized in *Table 1*. Differences in numbers of cases and controls were due to differences in quality of DNA in the samples. SNPs that could not be genotyped after three PCR tests were excluded.

**Table 1:** Genotype distributions in bacterial meningitis survivors versus controls.

SNP		Total BM	MM	PM	Controls
		n (%)	n (%)	n (%)	
		Total 473	Total 391	Total 83	
***TLR2*** ** +2477**		466	384	82	1141
	**GG**	418 (89.6)	345 (89.8)	73 (89.1)	1041 (91.2)
	**GA**	46 (9.9)	37 (9.6)	9 (10.9)	96 (8.4)
	**AA**	2 (0.5)	2 (0.6)	0 (0.0)	4 (0.4)
	***P*** **-value^1^**	1.0	0.6	1.0	
	**OR (95% CI)**	1.2 (0.2–6.7)	1.5 (0.3–8.1)	1.5 (0.1–29.0)	
***TLR4*** ** +896**		456	376	80	1141
	**AA**	401 (87.9)	328 (87.2)	73 (91.2)	1001 (87.7)
	**AG**	41 (9.0)	36 (9.6)	5 (6.3)	136 (11.9)
	**GG**	14 (3.1)	12 (3.2)	2 (2.5)	4 (0.4)
	***P*** **-value^1^**	1.1*10^−5^	1.2*10^−5^	0.05	
	**OR (95% CI)**	9.0 (2.9–27.5)	9.4 (3.0–29.2)	7.3 (1.3–40.4)	
***NOD1*** ** +32656**		450	372	78	1141
	**AA**	260 (57.8)	210 (56.5)	50 (64.1)	663 (58.1)
	**AC**	161 (35.8)	136 (36.6)	25 (32.1)	414 (36.3)
	**CC**	29 (6.4)	26 (6.9)	3 (3.8)	64 (5.6)
	***P*** **-value** 2	0.8/0.5	0.3/0.3	0.5/0.8	
	**OR (95% CI)**	1.2 (0.7–1.8)	1.3 (0.8–2.1)	0.7 (0.2–2.2)	
***NOD2*** ** SNP8**		463	381	82	1141
	**CC**	414 (89.4)	341 (89.5)	73 (89.0)	1063 (93.2)
	**CT**	41 (8.9)	32 (8.4)	9 (11.0)	76 (6.7)
	**TT**	8 (1.7)	8 (2.1)	0 (0.0)	2 (0.1)
	***P*** **-value^1^**	0.001	0.0004	0.2	
	**OR (95% CI)**	10.0 (2.1–47.4)	12.2 (2.6–57.8)	1.7 (0.8–3.5)	
***NOD2*** ** SNP12**		454	379	75	1141
	**GG**	443 (97.6)	369 (97.4)	74 (98.7)	1096 (96.1)
	**GC**	8 (1.8)	8 (2.1)	0 (0.0)	45 (3.9)
	**CC**	3 (0.6)	2 (0.5)	1 (1.3)	0 (0.0)
	***P*** **-value^1^**	0.02	0.06	0.06	
	**OR (95% CI)**	17.7 (0.9–344.0)	15.1 (0.7–316.0)	46.0 (1.9–1139.0)	
***NOD2*** ** SNP13**		461	381	80	1141
	**−/−**	442 (95.9)	365 (95.8)	77 (96.2)	1079 (94.6)
	**−/C**	18 (3.9)	15 (3.9)	3 (3.8)	62 (5.4)
	**C/C**	1 (0.2)	1 (0.3)	0 (0.0)	0 (0.0)
	***P*** **-value^1^**	0.3	0.2	NA	
	**OR (95% CI)**	7.4 (0.3–183.0)	9.0 (0.4–222)	NA	
***CASP1 -*** **8404**		469	388	81	1140
	**AA**	281 (59.9)	231 (59.5)	50 (61.7)	650 (57.0)
	**AG**	156 (33.3)	132 (34.0)	24 (29.6)	414 (36.3)
	**GG**	32 (6.8)	25 (6.5)	7 (8.7)	76 (6.7)
	***P*** **-value** 2	0.9/0.9	0.9/1.0	0.5/0.5	
	**OR (95% CI)**	1.0 (0.7–1.6)	1.0 (0.6–1.5)	1.3 (0.6–3.0)	

^1^ Fisher's exact test.

2 Chi^2^/Fisher's exact test.

SNP: single nucleotide polymorphism, BM: bacterial meningitis, MM: meningococcal meningitis, PM: pneumococcal meningitis, OR: Odds Ratio, 95% CI: 95% confidence interval, NA: not applicable.

Different numbers in cases are due to different quality of DNA.

*P*-values and ORs were calculated for homozygous mutant alleles versus WT and heterozygous alleles.

Genotype frequencies of BM survivors were compared to those in controls and MM and PM patients were also separately compared to controls.

Carriage of homozygous mutant alleles for *TLR4* +896 predisposed to susceptibility to develop BM. Significantly more BM patients than controls were affected (*p* = 1.1*10^−5^, odds ratio (OR) 9.0, 95% confidence interval (CI) 2.9–27.5). This was even stronger for MM patients compared to controls (*p* = 1.2*10^−5^, OR 9.4, 95% CI 3.0–29.2). For PM patients the difference was not statistically significant.

Significant differences in genotype frequencies were also found for *NOD2* SNP8 when comparing carriage of homozygous mutant alleles with heterozygous or homozygous wild types in the total group of BM patients (*p* = 0.001, OR  = 10.0, 95% CI  = 2.1–47.4) which was no longer significant after correction for multiple testing. MM patients also carried more often homozygous mutant alleles of *NOD2* SNP8 than controls (*p* = 0.0004, OR  = 12.2, 95% CI  = 2.6–57.8). The difference between PM patients and controls was not statistically significant.

For the other tested SNPs we did not find differences in genotype frequencies comparing patients to controls.

### Carrier trait analysis

*Table 2* shows the traits significantly associated with susceptibility to BM.

**Table 2:** Significant results of carrier trait analyses.

		Total BM	MM	PM	Controls
SNP combination	Genotypes	n (%)	n (%)	n (%)	n (%)
***TLR4*** ** +896/** ***TLR2*** ** +2477**	GG/AA	13 (2.9)^2^	11 (2.9)	2 (2.5)	4 (0.4)
	All other alleles	440 (97.1)	362 (97.1)	78 (97.5)	1137 (99.6)
	P-value^1^	3.4*10–5	4.17*10–5	0.05	
	OR	8.4	8.6	7.3	
	95% CI	2.7–25.9	2.7–27.3	1.4–40.4	
**TLR4 +896/NOD2 SNP8**	GG/TT	12 (2.7)^2^	10 (2.7)	2 (2.5)	3 (0.3)
	All other alleles	437 (97.3)	359 (97.3)	78 (97.5)	1138 (99.7)
	P-value^1^	2.8*10–5	4.15*10–5	0.04	
	OR	10.4	10.6	9.7	
	95% CI	2.9–37.1	2.9–38.6	1.6–59.1	

^1^ Fisher's exact test.

2 The observed frequency of combined carriage (*i.e.* intersection) of the homozygous mutant alleles is relatively high compared the expected frequency (*i.e.* the frequency of one genotype multiplied by the other genotype; see table 1). The genotypes were retested and confirmed, and treated as empirical data.

Abbreviations: SNP: single nucleotide polymorphism, BM: bacterial meningitis, MM: meningococcal meningitis, PM: pneumococcal meningitis, OR: Odds ratio, 95% CI: 95% confidence interval.

Combined carriage (*i.e.* the intersection) of homozygous mutant alleles *TLR2* +2477 and *TLR4* +896 strongly enhanced the predisposition to develop BM (*p* = 3.4*10^−5^, OR  = 8.4, 95% CI  = 2.7–25.9). This effect was even stronger for MM patients compared to controls (*p* = 4.2*10^−5^, OR  = 8.6, 95% CI  = 2.7–27.3), however for PM it was not statistically significant. We also found a significant trait with *TLR4* +896 and *NOD2* SNP8. The combination of these SNPs when carrying both homozygous mutant alleles (*i.e.* the intersection of both SNPs) showed a strong association with BM, most pronounced for MM (for BM *p* = 2.8*10^−5^, OR  = 10.4, 95% CI  = 2.9–37.1 and for MM *p* = 4.2* 10^−5^, OR  = 10.6, 95% CI  = 2.9–38.6 and not significant for PM). The observed frequency of simultaneous carriage (*i.e.* intersection) of the homozygous mutant alleles in the *TLR2* +2477/*TLR4* +896 and *TLR4* +896/*NOD2* SNP8 combinations is relatively high compared the expected frequency (*i.e.* the frequency of one genotype multiplied by the other genotype; see table 1). The genotypes were retested and confirmed, and therefore treated as empirical data.

Other traits with *TLR4* or *NOD2* SNPs did not show a combined effect. We also could not identify significant associations when combining the other SNPs.

## Discussion

Comparing genotype frequencies between BM survivors and healthy controls we showed that *TLR4* +896 and *NOD2* SNP8 were significantly associated with susceptibility to develop MM. The combined carriage of *TLR2* +2477 and *TLR4* +896 mutants as well as the combination of *TLR4* +896 and *NOD2* SNP8 mutants were identified as genetic traits significantly associated with susceptibility to develop MM. Our results were highly significant and were robust after correction for multiple testing. Associations in the PM patient group showed trends in concordance with the results for the MM patients, but a higher number of patients is needed to study the role of these SNPs in PM.

Our study is the first to associate *NOD2* with susceptibility to MM, both in single-and multigene analyses. NOD2 is an intracellular PRR containing a caspase-recruitment domain (CARD). *NOD2* SNPs are associated with inflammatory bowel disease and share a signaling defect in response to both the Gram-negative cell wall component lipopolysaccharide (LPS) as well as PGN in human experimental studies [20]. Mutant alleles of *NOD2* were associated with decreased activation of NFkB [21]. *In vitro* studies on murine microglia and astrocytes showed that NOD2 is expressed and upregulated by these cells after exposure to *N. meningitidis* [22], [23]. Experimental studies have shown that *in vitro* inflammatory responses of both murine astrocytes and microglia are significantly reduced in the absence of NOD2 after stimulation with *N. meningitidis.* Astrogliosis, demyelination, behavioral changes, and increased inflammatory cytokine levels within the CNS in meningococcal infection are all reduced in *NOD2* knockout mice [14]. Both the human and mice data indicate that NOD2 represents an important component in the generation of damaging CNS inflammation following meningococcal infection [14].

Although murine data might correlate poorly with some human conditions [24] they are often used for modeling, hypothesis testing, and disease linkage to chromosomal regions and genes, as has been done in many cases including ulcerative colitis and the identification of hyporesponsiveness to LPS by identifying *TLR4* mutations [25]–[27].

The role of TLRs in CNS infection is well recognized and consists of a combination of specific responses to the causative pathogen and also of non-specific activation of the innate immune system [28]. Although synergistic effects for TLR2 and TLR4 have been described for tuberculosis, malaria, and lupus, our study is the first to associate a genetic trait for *TLR2* and *TLR4* SNPs with susceptibility to meningitis [29]. Although TLR2 and TLR4 share the downstream MyD88 pathway resulting in NFkB transcription, it is also known that TLR2 and TLR4 triggering results in differential patterns of gene expression [30]. Toll-interleukin 1-domain-containing adapter-inducing interferon-β (TRIF) is another class of adapter proteins involved in TLR signaling. TLR4 activation results in the recruitment of both MyD88 and TRIF, whereas TLR2 activation results in the recruitment of MyD88 and not TRIF. MyD88 and TRIF are thought to orchestrate separate intracellular pathways because of temporal differences in how they activate NFkB [30]. Synergy between TLR2 and TLR4 activation has also been described in murine macrophages upon stimulation with LPS in the production of TNF-α [31], [32].

The combination of *TLR4* and *NOD2* SNPs was also strongly associated with susceptibility to develop MM. At first sight, this combination might not seem very comprehensive considering that TLR4 is a plasma membrane PRR and NOD2 is a cytosolic PRR. However, *TLR4* and *NOD2* were significantly associated with susceptibility to Crohn's disease in children in single gene analysis and gene-gene interactions [33]. Carriage of *TLR4* +896 mutants enhances the susceptibility to develop MM in the single gene analysis of our study. TLR4 recognizes LPS in the outer membrane of *N. meningitidis. TLR4* +896 mutant alleles are responsible for hyporesponsiveness to LPS in mice and humans in experimental studies [34], [35]. The same *TLR4* SNP has been associated with enhanced susceptibility to Gram-negative infections in adult surgical ICU patients compared to healthy volunteers [36]. In a cohort of children with invasive meningococcal infections *TLR4* +896 was correlated with mortality, increased frequencies of ventilation support, application of inotropic substances, skin grafting, and limb loss [37]. The proposed mechanism in both studies is impaired TLR4 mediated LPS responses with decreased pro-inflammatory intracellular signaling. However, in a retrospective case-control study of 252 Gambian children with serogroup A meningococcal meningitis (of which 120 were culture proven), no association was found with *TLR4* +896 and susceptibility to MM [38]. This difference may be due to the fact that 86% of our cohort consisted of serogroup B patients with only one case of serogroup A. Comparing the ability of distinct serogroups meningococci to stimulate PRRs could be an interesting focus for further research.

In order to validate the associations described in our study, these data should be replicated in another independent cohort. Ideally peers exposed to the same environmental factors should be used as controls. However, we consider our control cohort as valid since we used a large cohort of Dutch, ethnical matched controls, representative for the Dutch population. During the period of patient inclusion from 1999 to 2001, all controls were living in the Netherlands and incidence varied from 7–8/100.000 inhabitants [39]. Another limitation of this study is the retrospective design. We could not include DNA analysis of fatal cases of BM. However, including fatal cases of meningitis as well will provide interesting additional information to study the associations between SNPs and meningitis susceptibility. *TLR4* and *NOD2* SNPs were found to deviate from HWE. However, these SNPs were retained in the analysis since they all concern cases. Deviations in case genotype frequency can be an indication of association [40]. Although we used large case and control cohorts, *TLR4* and *NOD2* mutant alleles are rare, as seen by large confidence intervals. This may be due to selection pressure because of the possible adverse effect of these SNPs. Bigger cohorts should be tested and combined with other studies, also in different ethnical populations.

The relevance of identifying genetic variation predisposing for MM development is that it provides better understanding of the details of MM pathogenesis. It also enables the prediction of the individual risk to develop BM and might identify patients at high risk for severe disease and sequelae who need a costume-fit treatment and follow-up. Secondly, this knowledge may be implemented in clinical practice, for example the identification and targeted vaccination of high susceptible people. Another option is to add genetic severity risk factors to existing prediction models for hearing loss and academic or behavioral limitations after surviving BM [17], [41], [42]. In the future, this process might be fueled by the field of Public Health Genomics, involved in this translation and “the responsible and effective translation of genome-based knowledge and technologies into public policy and health services for the benefit of population health” (Bellagio statement, 2005: see www.graphint.org for details) [43], [44].

## Conclusions

In this study we show for the first time that *TLR4* +896 and *NOD2* SNP8 were strongly associated with susceptibility to develop MM in a single SNP analysis. Besides, we identified two genetic carrier traits. Simultaneous carriage of *TLR2* and *TLR4* SNPs and of *TLR4* and *NOD2* SNPs showed an even more pronounced association with susceptibility to develop MM. These data contribute to the current understanding of bacterial meningitis and may, in the future, help the identification of people at risk to develop severe infectious diseases, such as MM.
